# Towing icebergs to arid regions to reduce water scarcity

**DOI:** 10.1038/s41598-022-26952-y

**Published:** 2023-01-07

**Authors:** Alan Condron

**Affiliations:** Department of Geology and Geophysics, Woods Hole Oceanographic Institution, Woods Hole, MA 02543 USA

**Keywords:** Cryospheric science, Climate and Earth system modelling, Climate-change mitigation

## Abstract

Expanding agriculture, rising global population, and shifts in climate are placing increasing demands on existing water resources, especially in regions currently experiencing extreme drought. Finding new and reliable water sources is an urgent challenge. A long-held idea is that icebergs could be towed to arid coastal regions and harvested to help alleviate water stress. Here, a numerical model is used to simulate the deterioration of icebergs towed to Cape Town, South Africa and the United Arab Emirates (UAE). Moved at a speed of 0.5 m/s, an iceberg able to reach Cape Town must be at least ~ 300 m long and ~ 200 m thick at its time of capture. An iceberg this size would only require ~ 1 to 2 vessels to move and would deliver ~ 2.4 million liters of water. Placing an insulating material around the same iceberg to inhibit wave-induced erosion results in 4.5 billion liters of deliverable water. To reach the UAE, an unprotected iceberg needs to be at least ~ 2000 m long and 600 m thick, or 1250 m long and 600 m thick if insulated from wave-induced erosion. Icebergs of these dimensions would require ~ 10 to 20 vessels to move. Results are discussed in terms of the size and number of icebergs needed to help alleviate drought. In theory, small icebergs can easily be moved to South Africa; the challenge is likely to be harvesting the water as icebergs left offshore in a subtropical environment melt after a few days to weeks.

## Introduction

The idea of towing icebergs long distances to arid regions to help alleviate drought has a flamboyant history stretching back more than 200 years and is a topic that often draws the attention of the mainstream media^1–4^. There has also been an increasing number of scientific publications authored on this topic in recent years^5–8^. From what has already been written it appears that at the turn of the nineteenth century small icebergs were towed from Laguna San Rafael (~ 45°S) to Valparaiso, Chile (~ 33° S), and Callao, Peru (~ 12°S) for refrigeration. The idea of using icebergs as a source of drinking water, however, is most often credited to a seminar John Isaacs gave at the Scripps Institution of Oceanography in 1949^1,9,10^. Several decades later a series of technical papers appeared on this subject^11–13^ and soon after, the first *Iceberg Utilization* conference was held at Iowa State University^14^. In the concluding remarks of this meeting, it was noted that “icebergs should not be dismissed as a possible fresh water resource, and that further work is required to explore this possibility”.

Historically, attention has focused on towing icebergs to Saudi Arabia and the United Arab Emirates (UAE)^14^. However, the 2018 water shortage in Cape Town, South Africa, and narrow avoidance of what became known as ‘Day Zero’ – a time when the water level of the major dams supplying the city would fall below 13.5% – moved this idea back into consideration, especially given the relatively short distance from Antarctica to South Africa^2–4,6,15^. Most recently, the 2021 World Economic Forum listed harvesting icebergs in its top five alternative methods for alleviating water stress.

But while icebergs are routinely towed short distances to avoid collisions with offshore oil and gas platforms operating near the Grand Banks of Newfoundland, Canada, tows moving icebergs that are large enough to help alleviate water stress, over distances of several thousand kilometers, have never been performed, and many questions remain over the feasibility of undertaking them.

Here, a numerical iceberg model is used to simulate the deterioration of icebergs towed from Antarctica to Cape Town, South Africa, and to the United Arab Emirates, based on routes proposed by Winter^3^; Sloane^15^; and Alshehi^16^. Results are used to assess how large an iceberg must be to survive the tow and how many icebergs would need to be delivered to help alleviate water stress. This latter assessment is based on a series of metrics that range from providing a basic water intake for human survival, to supplying a certain percentage of a region’s usage.

##  Model setup

The MITberg iceberg model was used to study iceberg towing^17^. Rates of iceberg melt were derived by calculating ice loss caused by solar radiation, sensible heating, wave-induced erosion, and buoyant vertical convection (See Methods). As a first step to quantifying the overall accuracy of the model to the application of towing, a control simulation with realistic volumes of ice calved from the Antarctic ice sheet was performed (See Methods). Results shown in Figure S2 illustrate that this model can produce both an iceberg drift pattern and total ice volume in the Southern Ocean that is in good agreement with observations, thus demonstrating that it can be used to estimate ice loss during towing.

The towing experiments were performed at speeds of 0.25 m/s and 0.5 m/s (0.5 and 1 knot;^12^) and assumed the towing vessel could overcome all external drag forces exerted on the iceberg by the ocean and atmosphere. Initial experiments were performed assuming no protective material was placed around or over the iceberg to reduce melt (termed ‘unprotected’), while a second set of simulations assessed improvements in ice survival when an insulating material was placed around the iceberg at the waterline. The main results discussed in this manuscript are based on the iceberg tow beginning at the end of the austral spring/start of the austral summer (taken to be 1st December in the model experiments) given that longer hours of daylight, lower wind speeds, and reduced ocean wave heights, would make capturing an iceberg less hazardous. However, to assess the importance of the start date on the volume of deliverable ice, a second set of experiments towing an iceberg to Cape Town starting the model at the beginning of the austral winter (1st May) were performed. In each case, the deterioration of icebergs ranging in length from 200 m – 3000 m and in thickness from 150 – 600 m (see Suppl. Table 3) was studied.

**Figure 1:**
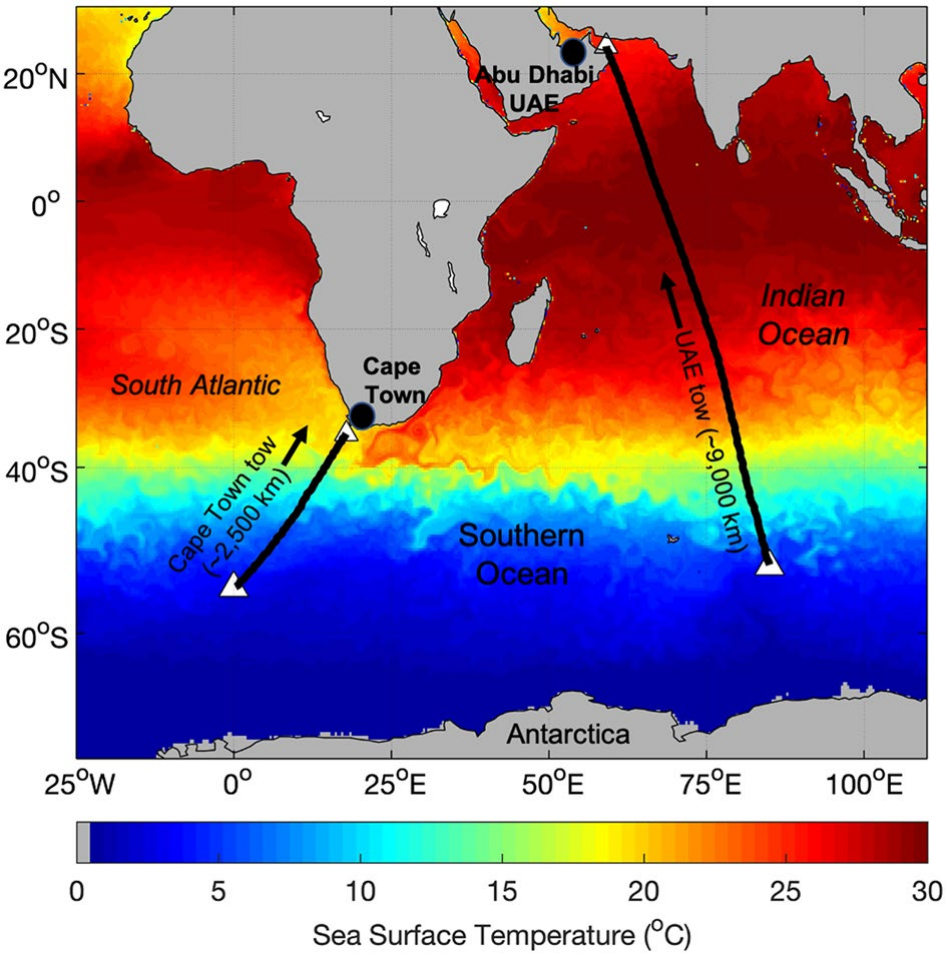
Simulated iceberg tow routes. Antarctic icebergs were towed from 55°S,0° W to Cape Town, South Africa [~ 2500 km] and from 52°30′ S, 85° E to the United Arab Emirates (UAE) [~ 9000 km]. The blue-red colours show a snapshot of model simulated sea surface temperature (SST) for the month of April to illustrate that in order to reach these destinations, icebergs must be moved through subtropical-tropical (20 °C +) waters for long periods of time.

Both tow routes begin in the Southern Ocean (just south of 50°S) to capture icebergs that have naturally drifted away from the continent and are closer to their final destinations (Fig. 1). The two routes also start relatively close to small islands that could act as places to coordinate any real-life tows, i.e., Gough Island for the Cape Town tow, and Heard Island and McDonald Islands for the UAE tow. The South Africa route begins at 55°S, 0°W and involves pulling an iceberg ~ 2500 km in a northeasterly direction to Cape Town, along a route discussed in Sloane^15^ and Winter^3^ (Fig. 1). The UAE route starts at 52°30′ S, 85° E and involves moving an iceberg in a north–north-westerly direction for ~ 9000 km to the Persian Gulf (Fig. 1). For this route, the shallow depth (~ 70 – 100 m) of the Strait of Hormuz connecting the Gulf of Oman with the Persian Gulf prevents large icebergs being towed directly to the capital city of the UAE, Abu Dhabi. A more viable delivery location is Al-Fujairah, on the Gulf of Oman coast, where the water is much deeper. Water harvested from an iceberg in this region could then be piped to its required destinations in the UAE^16^. At tow speeds of 0.5 m/s and 0.25 m/s, it takes 59 – 118 days to pull an iceberg to Cape Town and 206 – 412 days for an iceberg to reach Al-Fujairah.

##  Results

**Figure 2:**
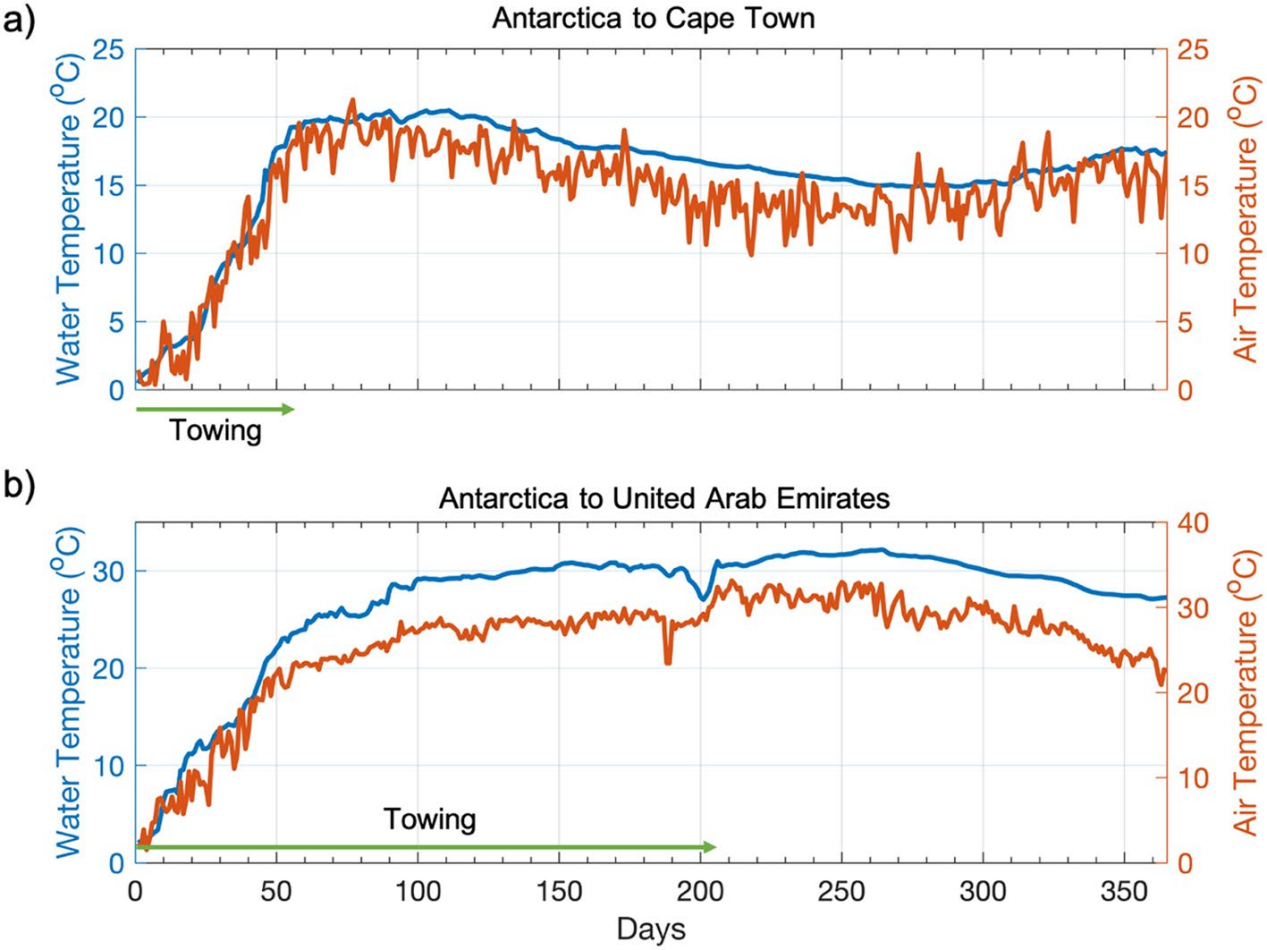
Ocean and atmospheric temperatures experienced by an iceberg towed from Antarctica to (**a**) Cape Town, South Africa, and (**b**) the United Arab Emirates. The green arrows represent the period during which the iceberg is being towed to its destination. At a tow speed of 0.5 m/s, an iceberg will reach Cape Town after 59 days and the United Arab Emirates after 206 days. In this figure the iceberg tows began on December 1st to correspond with the beginning of the austral summer.

Regardless of the season in which an iceberg is captured, icebergs moved from the Southern Ocean to the two destinations are subject to long periods when the environmental conditions create high rates of ice melt. At the start of both tows, air and water temperatures are close to the freezing point, and ice loss is minimal. After just one month of towing at 0.5 m/s an iceberg moving to either destination will cross the Polar and Sub Antarctic fronts and enter a region where water and air temperatures are ~ 10 °C (Fig. 2). As the tows continue north, temperatures also increase so that an iceberg arriving in Cape Town will constantly be surrounded by ~ 15 to 20 °C air and water temperatures. For the UAE tow, the environmental conditions are even more challenging as the iceberg must endure temperatures of ~ 25 °C – 30 °C for at least 100 + days as the ice moves through the Indian Ocean (Fig. 2). The iceberg arriving in the UAE will continue to be surrounded by similarly warm conditions, creating a substantial challenge for harvesting any iceberg before it entirely melts.

For the Cape Town route, the model indicates that the smallest iceberg able to survive being towed ~ 2500 km at 0.5 m/s would need to be 300 m long and 250 m thick at its point of capture. Icebergs of this size are very abundant in the Southern Ocean^18^, as well as in the model in this region (Figs. S3–S4). The long duration of the tow does however take a toll on the iceberg’s size. In this case, the iceberg would only be 16.3 m long and 15.7 m thick upon arrival, and contain < 1% of its initial volume, or ~ 2.4 million liters of water.

To assess if towing at a slower speed improves ice survival, the same set of experiments were performed moving icebergs at 0.25 m/s (Fig. 4; Fig. S6). The slower speed acts to reduce ice loss caused by sensible heating and wave-induced erosion as both are dependent on the relative velocity of the iceberg to the ocean and wind (see Methods), but halving the speed also doubles the duration of the tow so that icebergs are now in a warm subtropical environment for longer. Significantly, results from these experiments show that the same size iceberg surviving the faster 0.5 m/s tow would entirely melt 15 days before reaching Cape Town. The smallest iceberg capable of enduring this tow had to be ~ 500 m long 300 m thick, which is ~ 3-times the mass of the smallest iceberg surviving the faster tow.

While this result shows that towing an iceberg more quickly to its destination allows a smaller iceberg to initially be selected, a brief consideration of the force required to move an iceberg is necessary. To tow an iceberg 300 m long and 250 m thick at 0.5 m/s requires a bollard pulling force of ~ 6 × 10^6^ N (Newtons), while the larger 500 m long by 300 m thick iceberg towed at 0.25 m/s only requires a towing force of ~ 3 × 10^6^ N, i.e., half that needed to move the smaller iceberg faster (See Methods). One of the largest tug boats currently in operation, the Island Victory, has a bollard pulling power of ~ 4.7 × 10^6^ N. This vessel could move the 500 m long by 300 m thick iceberg at the required velocity of 0.25 m/s and, in theory, up to a speed of ~ 0.3 m/s. However, moving the smaller iceberg at 0.5 m/s exceeds the pulling power of this vessel by ~ 1.3 × 10^6^ N meaning an additional vessel would be required.

A second question to ask beyond an iceberg simply surviving the tow is “Will the delivered amount of water help alleviate water stress?” At a very basic level, research suggests that the average person requires 2.7–3.7 liters of water every day to remain healthy^19^. This means that the smallest iceberg surviving the 0.5 m/s Cape Town tow would provide ~ 650,000–890,000 people with water for a day, or ~ 16 to 22% of the cities ~ 4 million population^20^. If the delivered iceberg was to supply the basic drinking water demand of the entire population for one full-year (~ 10.8 to 14.8 million liters) then it would need to be ~ 700 m long and ~ 250 m thick at the beginning of the tow, if moved at 0.5 m/s. An iceberg with these dimensions moved at this speed requires a bollard pulling force of 1.4 × 10^7^, or ~ 3 tugs similar to the Island Victory. Upon arrival this iceberg would be ~ 390 m long and ~ 177 m thick and provide ~ 15.5 million liters of water. However, if the iceberg is left offshore while it is harvested, the model indicates that it will entirely melt after 44 days. Precisely how icebergs will be harvested remains a largely unanswered question, although ideas include open-cast mining, melting in a dry-dock, or breaking into smaller fragments^9,15^, but it needs to be done quickly.

**Figure 3:**
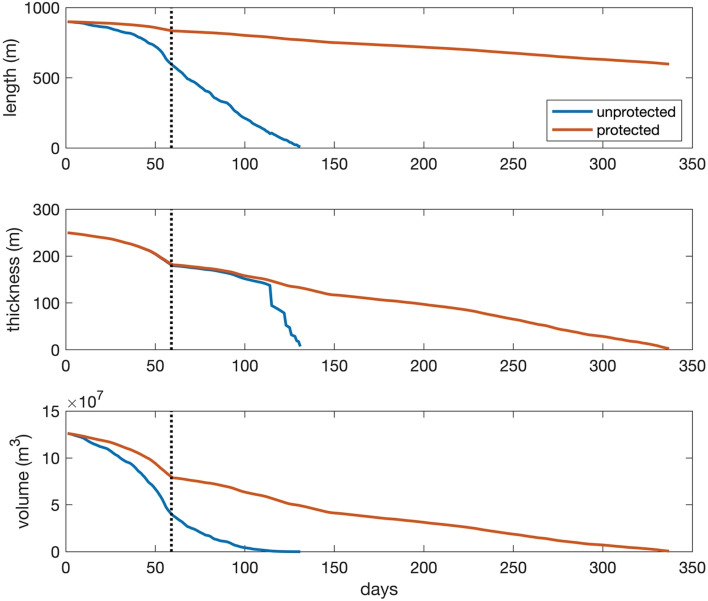
Deterioration of an iceberg 900 m long and 250 m thick towed from Antarctica to Cape Town, South Africa. *Protected* refers to experiments without wave-induced erosion. The iceberg was towed at 0.5 m/s. The vertical dotted lines denote the day the iceberg arrives at its destination.

There are other ways to assess the ability of icebergs to alleviate water stress. For example, it has been estimated that an iceberg might provide ~ 20% of Cape Town’s 500 million liter per day water demand for one-year (~ 36.5 billion liters per year) as this is equivalent to the amount of water provided by the Steenbras Lower Dam, one of six large dams that supply water to the Cape Town region^3,15^. To achieve this, the model indicates that an iceberg towed at 0.5 m/s would require an initial length and thickness of 900–1000 m and 200–250 m. An iceberg of this size would also retain ~ 30 to 35% of its original volume upon delivery (Fig. 3; Fig. S7). From an economic perspective, this iceberg would have a value of US$ 19.6 million – US$ 114.4 million, if compared to the costs of using desalination (US$ 0.49–2.86 per m^3^;^21^) to produce the same amount of water. This estimate does not, however, account for expenses associated with operating and maintaining the towing vessel(s) and, indeed, the final costs of performing an entire tow are particularly challenging to determine as they depend on the precise vessel(s) selected for the tow, cost of fuel, the actual route followed, and crew salaries^5,8^. While determining such numbers is beyond the scope of this research, the subject of economic viability will be addressed in forthcoming work.

Prior research has also given consideration to using engineering strategies to reduce ice melt by wrapping an insulating material around the iceberg or using a geotextile skirt at the waterline to reduce wave-induced erosion^15,22^. To assess how implementing such a technique might reduce ice loss, experiments setting wave-induced erosion to zero in the iceberg model were performed at tow speeds of 0.5 m/s. Remarkably, the model shows that without wave-induced erosion even the smallest iceberg in the iceberg dataset (200 m long by 150 m thick, Suppl. Table 3) was able to survive being towed to Cape Town. In fact, upon arrival it was still 100 m long and 51 m thick and provided ~ 328 million liters of water, roughly 10% of its original content. For comparison, the same size iceberg towed without protection from waves completely melted after ~ 50 days, ~ 390 km from the South African coast.

**Figure 4:**
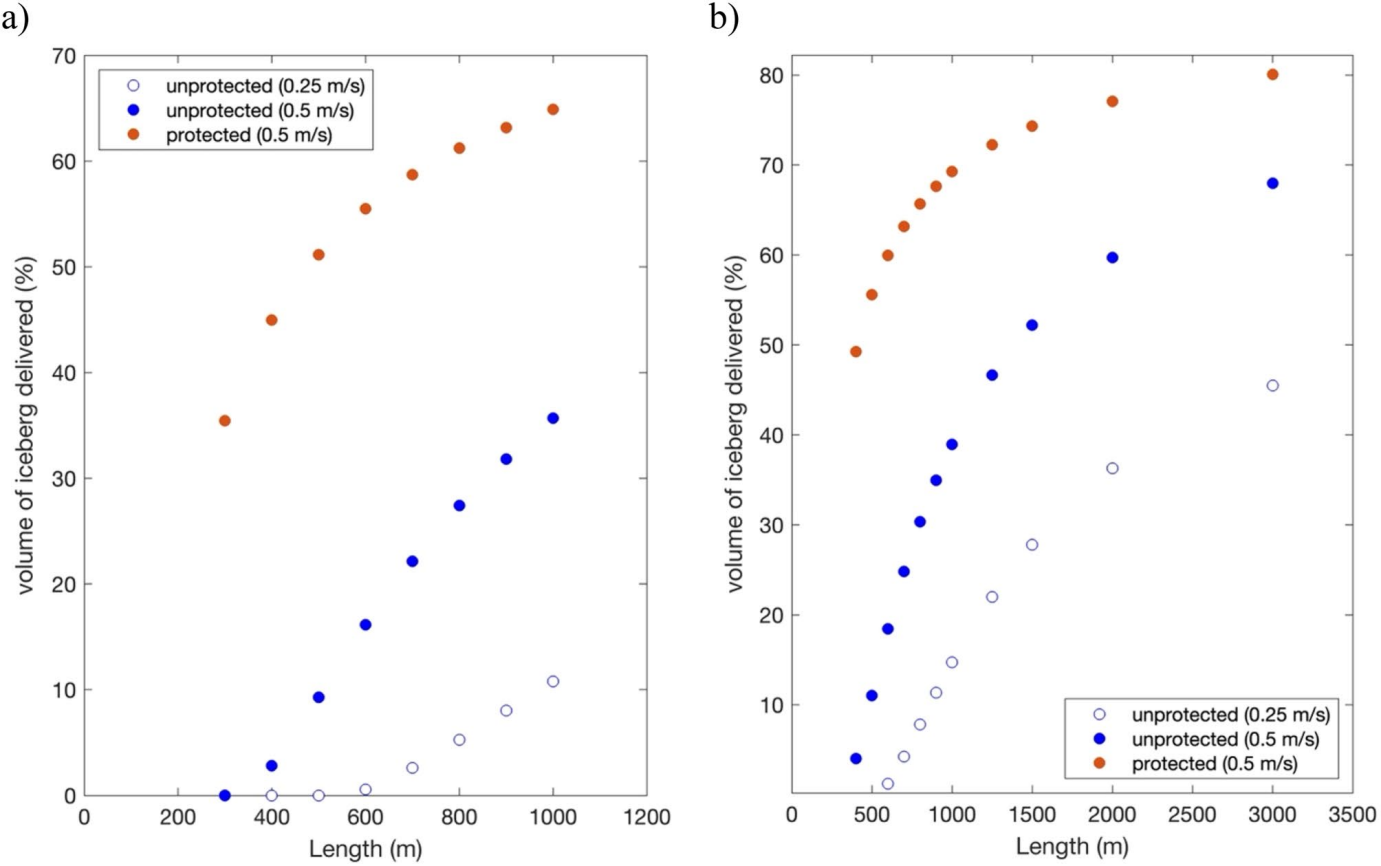
Percentage of deliverable ice to Cape Town, South Africa. Results are shown for 250 m thick (**a**) and 300 m thick (**b**) icebergs towed at 0.25 m/s and 0.5 m/s. *Protected* refers to experiments without wave-induced erosion.

To provide 20% of Cape Town’s annual water requirements, an iceberg protected from wave-induced erosion would have to be 700 m long and 225 m thick at its point of capture, i.e., half the weight of the unprotected iceberg. Further analysis of the impacts of wave-induced erosion also show that smaller icebergs benefit most from being insulated (Fig. 4). For example, an unprotected iceberg 300 m long and 250 m thick moved to Cape Town retains less than 1% of its volume, while insulating it results in 35% of its original water content being delivered. Figure 4 also shows there are also clear advantages to towing larger icebergs. For example, an unprotected 1000 m long iceberg 300 m thick will retain ~ 38% of its original volume, or 69% if towed protected from wave-induced erosion.

In order to assess the extent to which performing the tow at other times of the year changes the amount of deliverable ice, the Cape Town model experiment was started in May (using a towing velocity of 0.5 m/s) to simulate a tow performed during the austral winter. Interestingly, the results from this experiment indicate that, on average, slightly less ice (by a factor of ~ 0.8) arrives at its destination, compared to the summer tow. While this result may initially appear counterintuitive, an analysis of the environmental conditions experienced by the iceberg indicates that sea surface temperatures are actually several degrees warmer at the beginning of the tow (Fig. S5) as a result of ocean warmth persisting from the preceding austral autumn. In addition, higher wind speeds in the Southern Ocean during the winter months act to increase ice loss due to enhanced sensible heating and wave-induced erosion. While temperatures at Cape Town are cooler when the iceberg arrives (Fig. S5), this cooling is insufficient to offset the increased melt incurred during the tow.

In the final part of this manuscript, I discuss the feasibility of towing icebergs from Antarctica to the UAE. For this route, the smallest iceberg surviving the 206-day tow at a speed of 0.5 m/s initially had to be 2000 m long and 600 m thick. Upon arrival it was 312 m in length and 112 m thick. An iceberg with these dimensions holds 6.3 billion liters of drinking water which, by applying the same metrics as above, equates to enough water for 1.7 – 2.3 billion people for a day. As of 2021, the population of the United Arab Emirates was ~ 9.9 million^23,24^, meaning that this iceberg would (if all of its water was harvested) supply this region with a basic daily water intake for 189–255 days.

To move an iceberg this size at 0.5 m/s initially requires a bollard pulling force of 9.6 × 10^7^ N, or ~ 20 tugs rated similar to the Island Victory. Towing icebergs protected from wave-induced erosion provides a significant benefit, however. Of the different size icebergs examined, the smallest surviving the tow protected from wave-induced erosion had to initially be 1250 m long and 600 m thick, i.e., 40% of the weight of the unprotected iceberg, and would require ~ 12 tug boats to move. This iceberg also contains ~ 5-times more deliverable water (~ 31.8 billion liters).

The total water demand of the UAE is estimated at ~ 9000 billion liters per year. 24% (2230 billion liters) currently comes from desalination and this provides 100% of the countries domestic drinking water requirements, estimated at 1490 billion liters per year (~ 4 billion liters per day)^23^. An iceberg of the aforementioned size protected from wave-induced erosion could therefore provide ~ 7 to 8 days of the regions entire domestic water requirements, or just over one month if the iceberg water replaced 20% of that coming from desalination. An iceberg of this size would also have a value of US$ 17.0 million – US$ 99.3 million, based on the costs of desalinating the same volume of water.

## Conclusion

Results presented here show that both tow routes are theoretically feasible, although an iceberg surviving a tow to the Middle East (unprotected from wave-induced erosion) must be 2000 m in length and 600 m thick when the tow begins and would require ~ 10 to 20 vessels to move. Towing an iceberg to Cape Town is much more practical given the smaller size of the iceberg needed and the significantly fewer number of vessels needed to move it. The model also shows advantages to moving icebergs faster to their destination if the goal is to select the smallest iceberg able to survive the tow; a slower tow speed requires a larger iceberg to initially be selected, but does reduce the number of vessels needed to move it. Considerable gains in deliverable water are also made if wave-induced erosion can be reduced, especially for smaller icebergs. These results open up the possibility of developing a system where more than one iceberg is *en-route* to its destination at any one time to create a transit system where the arrival of a new iceberg continues to alleviate dependance on existing water sources such as groundwater, dams, and desalination.

## Methods

### Iceberg model (MITberg)

The iceberg model is coded in parallel FORTRAN and is capable of simulating the melt and drift of 10,000 s of icebergs in the ocean. Icebergs are assumed to be tabular, with a constant width (W) to length (L) ratio of 1:1.6. MITberg is coupled to the MITgcm ocean – sea ice model^25^, as described in Condron and Hill^17^. All experiments were performed using an eddy-permitting horizontal ocean resolution of 1°/6° (~ 18-km) with 50 levels in the vertical, ranging in thickness from 10 m near the surface to approximately 450 m at the maximum model depth. Sea ice is simulated using a dynamic-thermodynamic sea ice model that assumes a viscous-plastic ice rheology and computes ice thickness, ice concentration, and snow cover. The ocean–seaice–iceberg model was driven by solar radiation, precipitation, humidity, and winds from the ECWMF ERA40 atmospheric reanalysis data which has a 6-hourly temporal resolution and a global spatial resolution of 1.125° × 1.125°.

Iceberg deterioration (units: m/s) is from solar radiation, sensible heating, wave-induced erosion, and buoyant vertical convection. Melt from solar radiation, M_r_, reduces iceberg thickness as:

1 $$ M_{r} = \frac{{F_{sol} }}{{\rho_{i} \Gamma_{i} }}\left( {1 - \alpha } \right) $$

where *F*_*sol*_ is the solar radiation flux (W/m^2^) derived from the local downward and shortwave radiation flux, Γ_*i*_ is the latent heat of fusion of ice (J/kg) and α is the iceberg albedo (Table S1). Subaerial melt from sensible heating (also referred to as forced convection), *M*_*fa*_, is generated by the relative motion of the air passing the iceberg, and leads to both a reduction in waterline length and vertical thickness as:

2 $$ M_{fa} = \frac{{q_{f} }}{{\rho_{i} \Gamma_{i} }} $$

where *q*_*f*_ is the heat flux per unit surface area (W/m^2^),

3 $$ q_{f} = Nu k_{a} \Delta T/L $$

and *k*_*a*_ is the thermal conductivity of the fluid, Δ*T* is the difference between the local air temperature and the iceberg ($\Delta T = T_{a} - T_{i}$). The Nusselt number, *Nu*, gives the ratio of convective to conductive heat transfer as:

4 $$ Nu = 0.055 Re^{0.8} Pr^{0.4} $$

where the Reynolds number, *Re*, and Prandtl number, *Pr*, are defined as

5 $$ \begin{gathered} Re = \left| {v - v_{a} } \right|L/{\mathcalligra{v}}_{a} \hfill \\ Pr = {\mathcalligra{v}}_{a} /D_{a} \hfill \\ \end{gathered} $$

where ${\mathcalligra{v}}_{a}$ and *D*_*a*_ are the kinematic viscosity and thermal diffusivity of air, respectively. Melt is also generated by sensible heating below the waterline, *M*_*fw*_, and is calculated by replacing the constants for thermal conductivity, kinematic viscosity, and thermal diffusivity in Eqs. 8 and 10 with those for water (Table S1). Iceberg melt below the waterline from buoyant vertical convection, *M*_*l*_, along the side-walls reduces an icebergs width and length as follows:

6 $$ M_{l} = 8.82 \times 10^{ - 8} \Delta T + 1.5 \times 10^{ - 8} \Delta T^{2} $$

where Δ*T* is the difference between the ocean water temperature and the freezing point temperature of seawater. Finally, iceberg melt from wave erosion, *M*_*w*_, is simulated as:

7 $$ M_{w} = 0.000146\left( \frac{R}{a} \right)^{0.2} \left( {\frac{a}{{W_{p} }}} \right)\Delta T $$

where R is the roughness height of the iceberg and *W*_*p*_ the wave period (Table S3). The wave amplitude, *a*, is empirically related to wind speed and dependent on both sea ice fractional area and freeboard height, *Fb*, to avoid producing erroneously large wave drag forces. Finally, icebergs are considered to become unstable and roll-over when their length to thickness ratio is less than 0.7, (*L*/*T* < 0.7), and in this case, L and T are instantaneously swapped.

For the Southern Ocean iceberg experiments testing the validity of the model, ~ 3150 km^3^/yr of ice was discharged (as icebergs) from the Antarctic ice sheet from 53 locations, based on output from a modern-day ice sheet simulation^26^ (Fig. S2b). Ten different size icebergs were released from each calving margin (Supplementary Table 2). The model came into equilibrium after ~ 12 years (Fig. S1), with the model results plotted in Figs. S2–S4 based on output from years 12 to 25. Figure S2 show a good agreement between iceberg drift patterns in the model and observations. In addition, the simulated ice volume in the Southern Ocean (282.6 km^3^) is in remarkably good agreement with observational estimates (286.3 km^3^) produced by Tournadre et al.^18^.

Finally, the total bollard force, ${\vec{\text{F}}}_{b}$, required to move an iceberg at a required speed is calculated as:

8 $$ {\vec{\text{F}}}_{b} = \rho_{w} C_{d} Av^{2} $$

where *ρ*_*w*_ is the density of water, *A* is the area of the iceberg face perpendicular to the tow, *Cd* is a dimensionless form drag coefficient of value 1, and *v* is the towing velocity. Skin drag is neglected given the horizontal drag coefficient for water is 0.0012.

## Supplementary Information

Supplementary Information.

## Data Availability

The datasets used and/or analyzed in the current study are available from the corresponding author on request.
