# Supplementary material for: Towing icebergs to arid regions to reduce water scarcity
Source: Sci Rep. 2023 Jan 7;13:365. doi: 10.1038/s41598-022-26952-y (PMC9825379; doi:10.1038/s41598-022-26952-y)
Supplement: Supplementary file 1 — Supplementary Information. [file 41598_2022_26952_MOESM1_ESM.docx]

**Supplementary Material**

**Towing icebergs to arid regions to reduce water scarcity**

Alan Condron

Department of Geology & Geophysics, Woods Hole Oceanographic Institution,

Woods Hole, MA 02543

acondron@whoi.edu

**Figures**


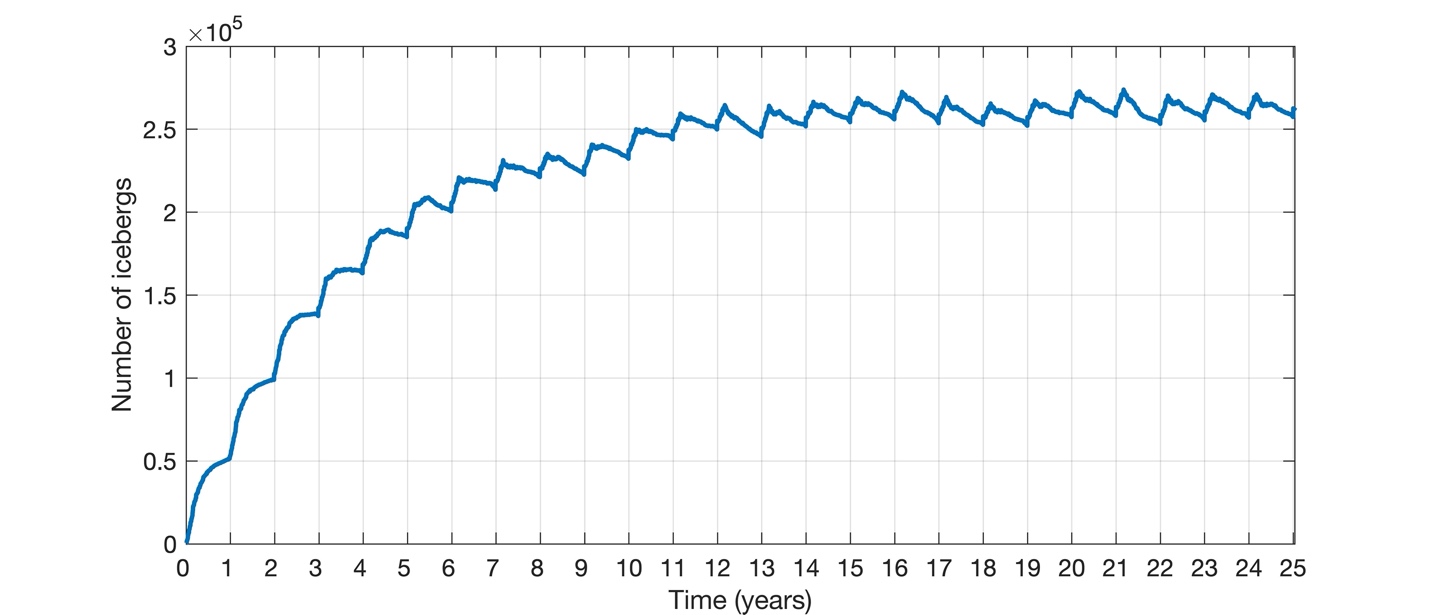


**Figure S1: Number of simulated icebergs in the Southern Ocean using the iceberg model, MITberg.** After 12 model years, the number of icebergs has come into equilibrium.


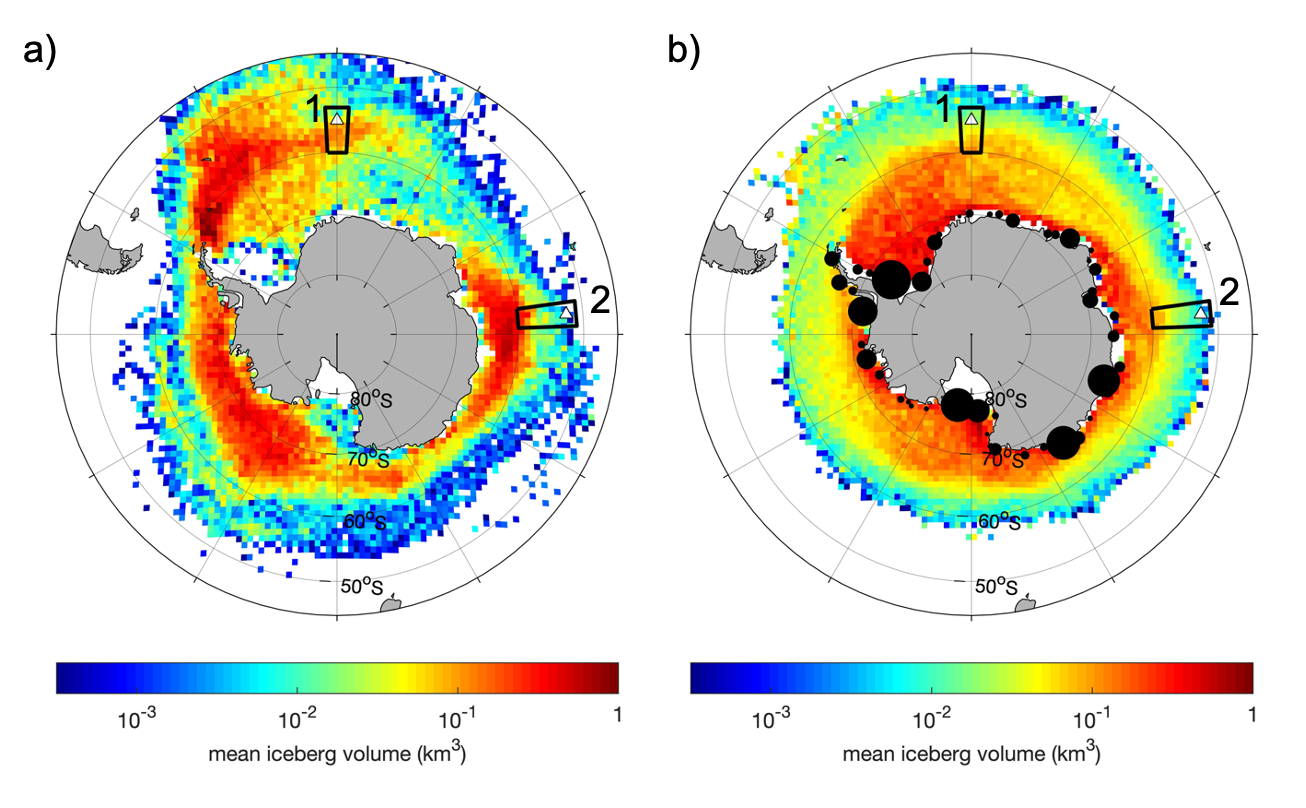


**Figure S2: Monthly mean iceberg volume in the Southern Ocean.** (a) Observational estimates based on satellite data from Tournadre et al., (2015), updated to cover the period 1992-2020, (b) simulated iceberg volume using MITberg. Black circles around the Antarctic coastline (panel b) denote the 53 locations and approximate flux of ice discharged from the continent at each location, based on a modern-day ice sheet model simulation (DeConto and Pollard 2016). The data in panel b have been regridded to the same 100x100km resolution as the satellite data to aid comparison. White triangles denote the start locations of the iceberg tows to (1) Cape Town, South Africa, and (2) the United Arab Emirates (UAE). Significantly, the simulated mean volume of ice in the Southern Ocean of 282.6 km^3^ agrees extremely well with observational estimates of 286.3 km^3^.


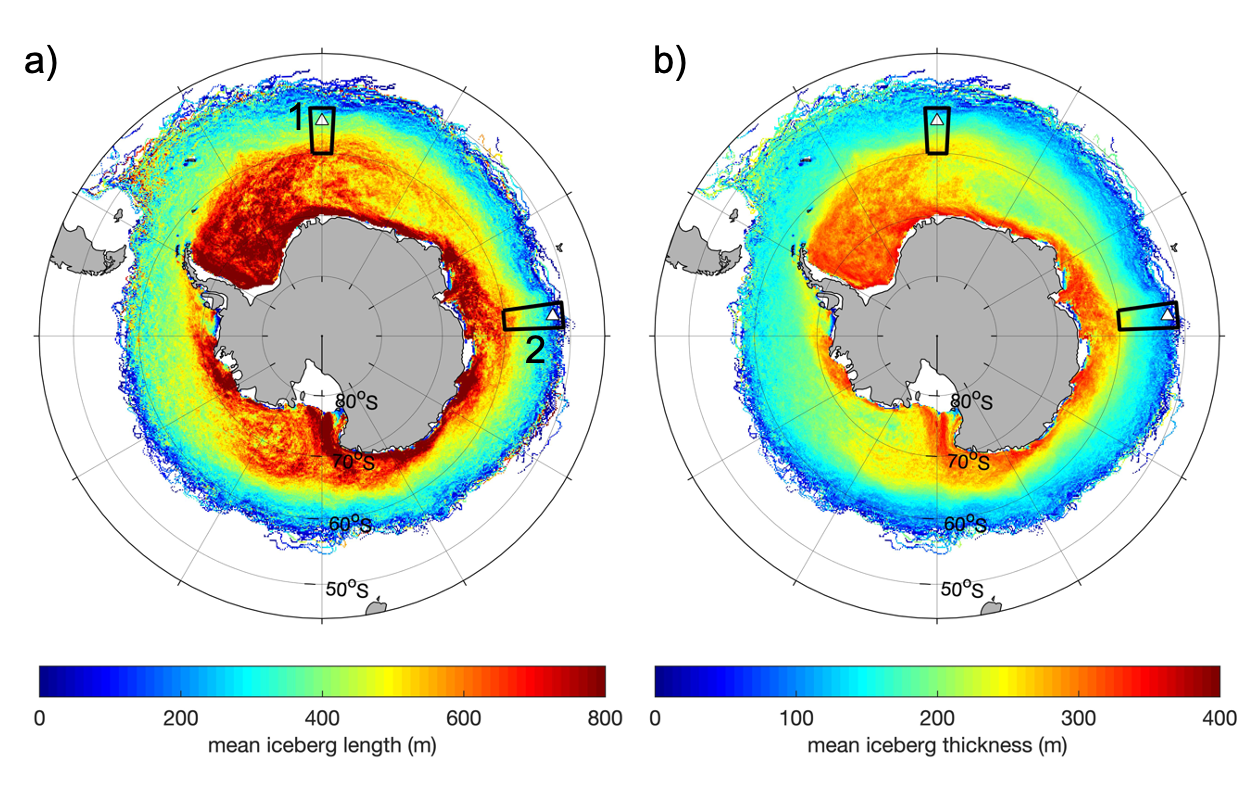


**Figure S3: Simulated monthly mean Southern Ocean iceberg (a) thickness and (b) length.** White triangles denote the start locations of the iceberg tows to (1) Cape Town, South Africa and (2) the United Arab Emirates (UAE).

| a)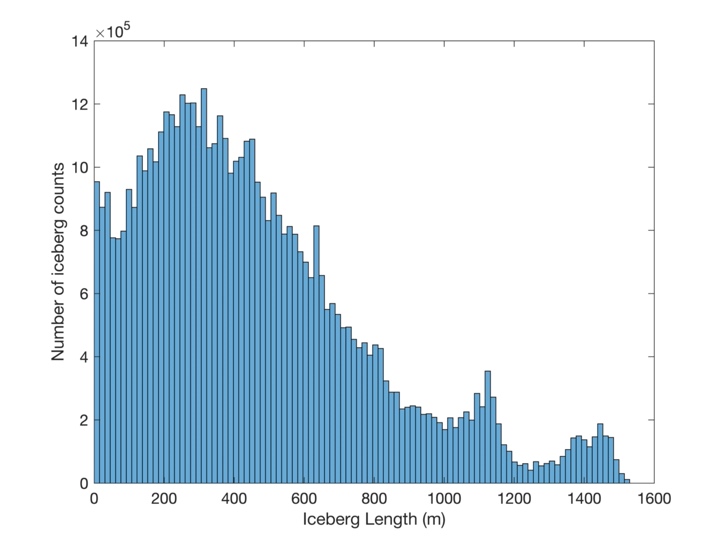 | b) 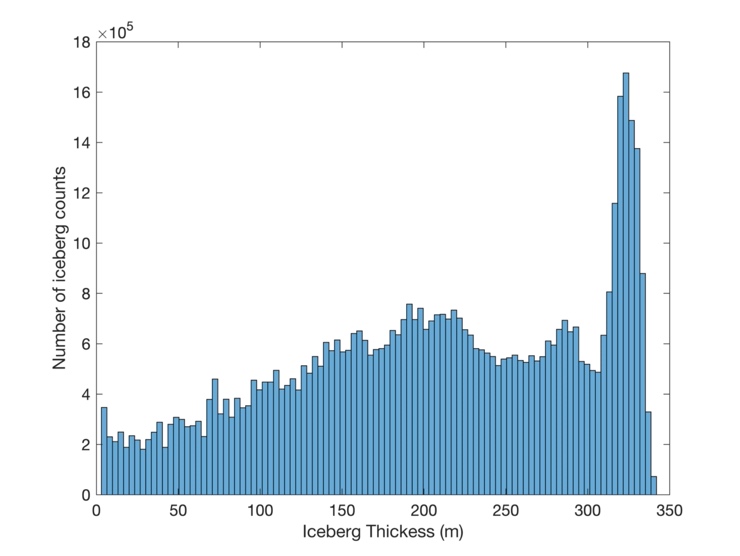 |
| --- | --- |
| c)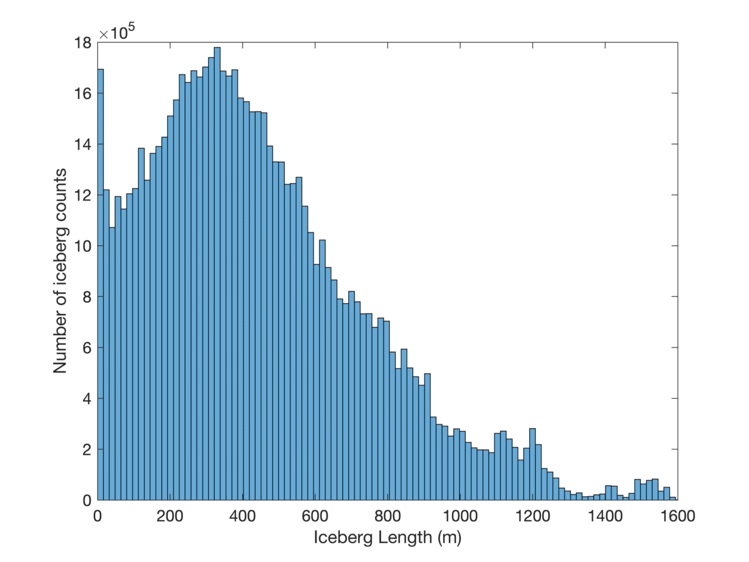 | d)  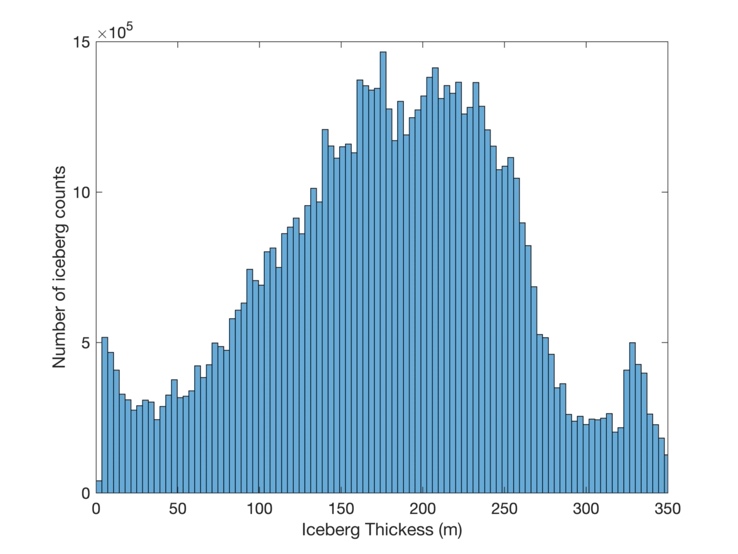 |

**Figure S4:** The size of icebergs found near to where the Cape Town (a-b) and UAE (c-d) tows begin. The data presented show icebergs length (a,c) and thickness (b,d) for the geographical regions 60°-53°S, 3°W-3°E (Cape Town tow) and 60°-51°S, 82°-88°W (UAE tow), as denoted by the black boxes in Figure S2-S3.

**
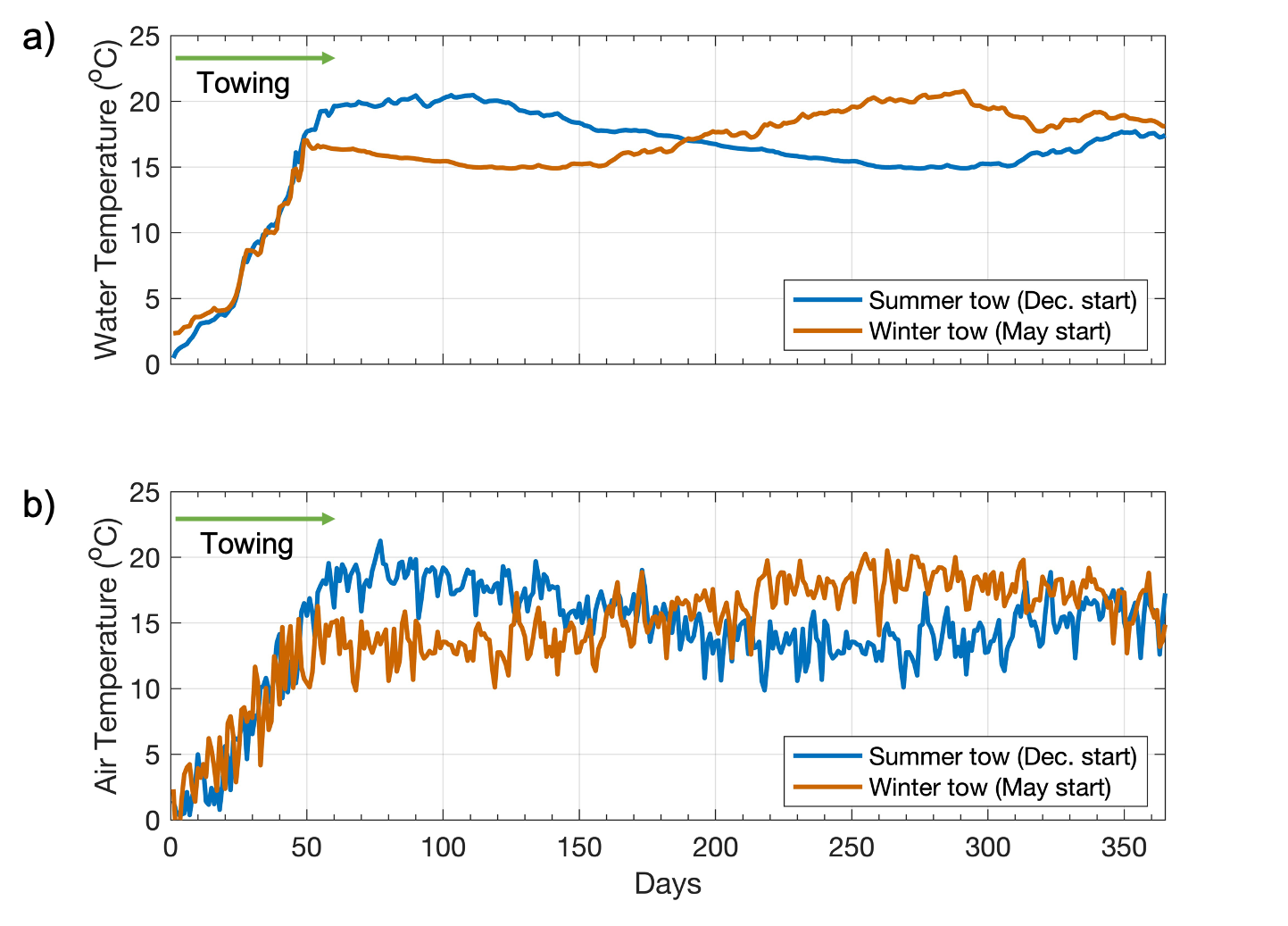
**

**Figure S5:** Ocean and atmospheric temperatures experienced by an iceberg towed from 55°S, 0°W to Cape Town, South Africa, during the austral summer (blue lines [tow starts: 1^st^ December] and austral winter (orange/red lines [tow starts 1st May]). The green arrows represent the 59 day period during which the iceberg is being towed (at a tow speed of 0.5 m/s) to its destination.

**
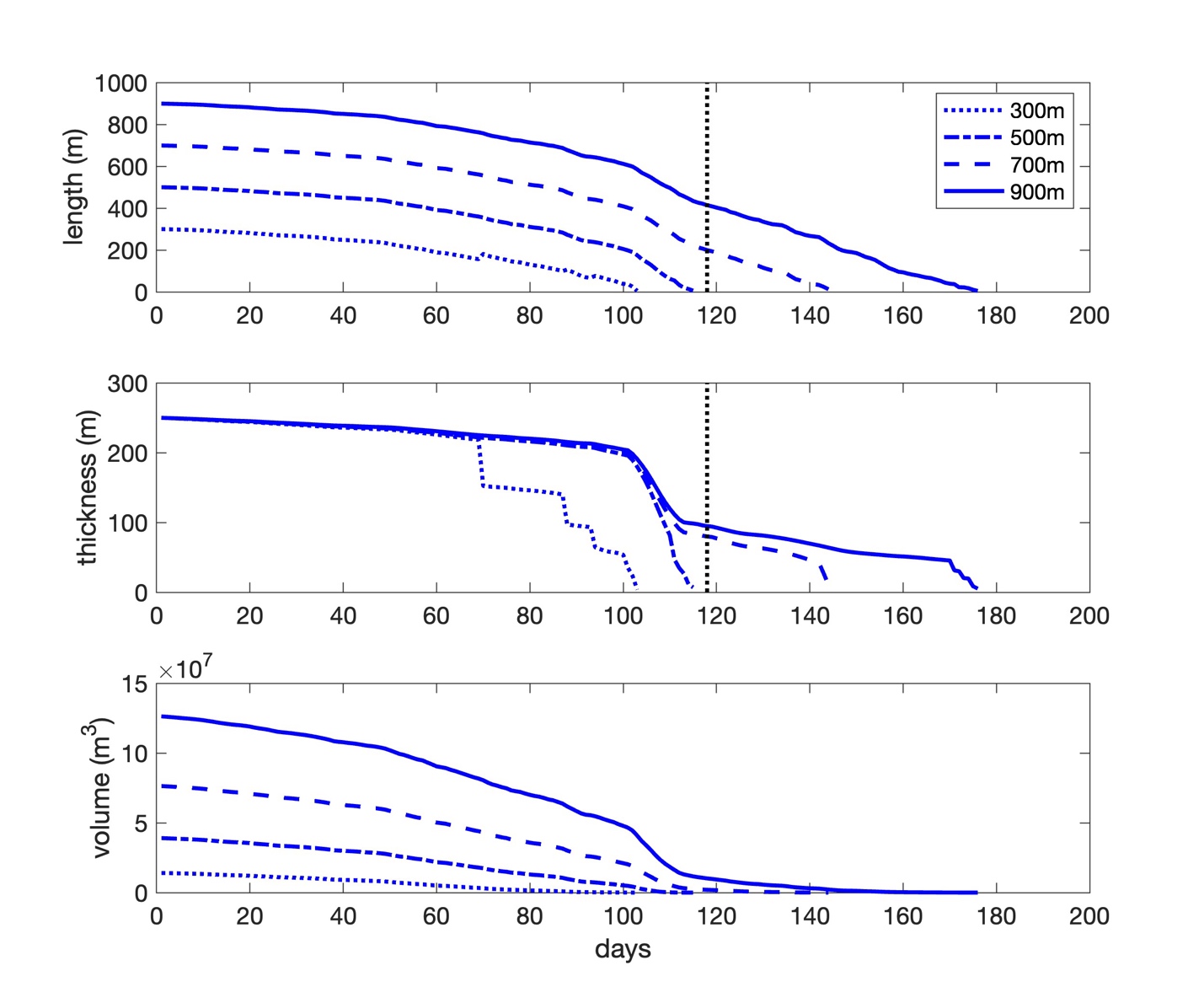
**

**Figure S6:** **Deterioration of icebergs towed from Antarctica to Cape Town, South Africa at a speed of 0.25 m/s.** The plots show changes in length, thickness and volume for icebergs initially 300m, 500m, 700m and 900m in length. Note that all icebergs were initially 250m thick. The vertical dotted lines denote the day the icebergs arrive at their destination.


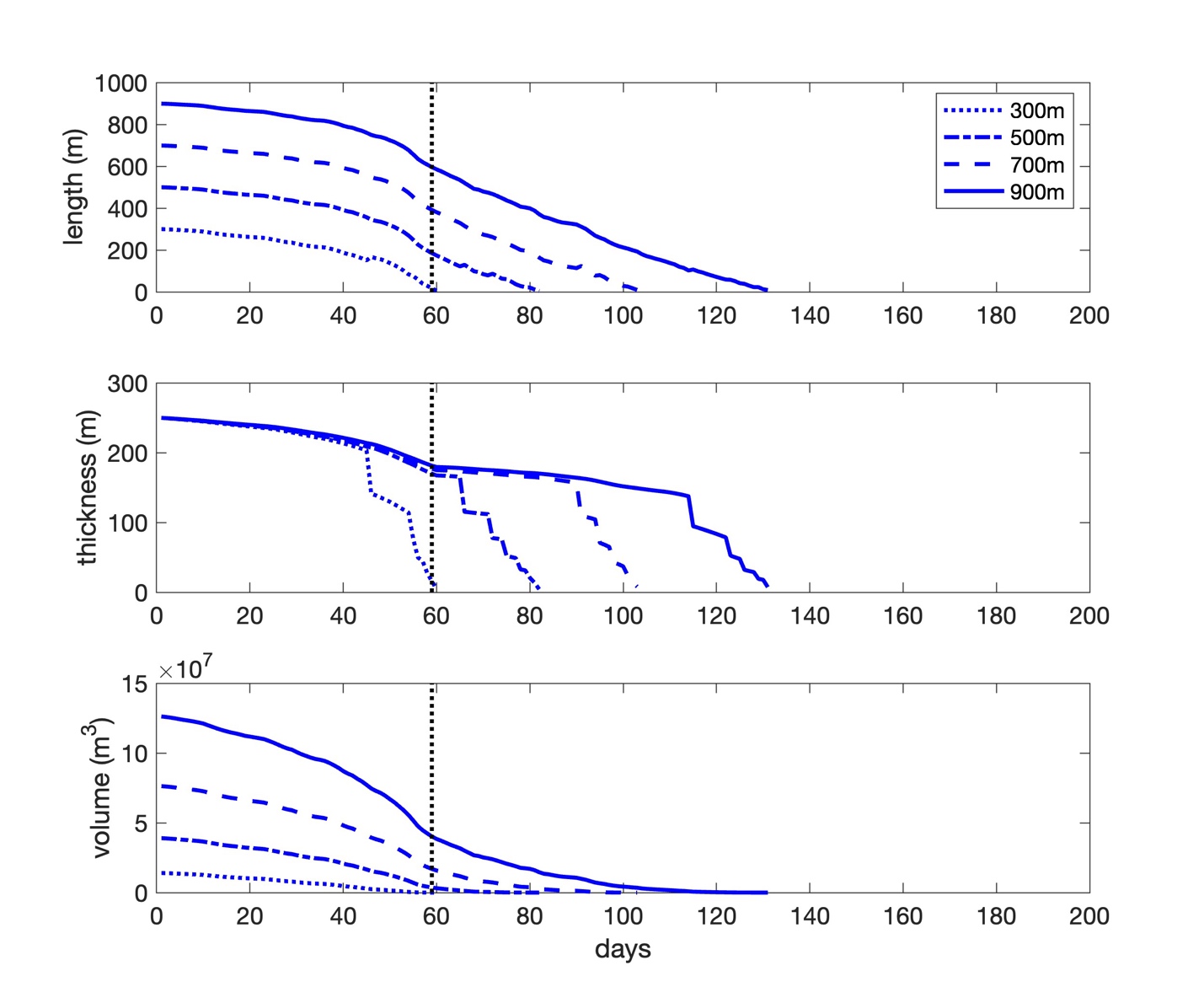


**Figure S7:** **Deterioration of icebergs towed from Antarctica to Cape Town, South Africa at a speed of 0.5 m/s.** The plots show changes in length, thickness, and volume for icebergs initially 300m, 500m, 700m and 900m in length. Note that all icebergs were initially 250m thick. The vertical dotted lines denote the day the icebergs arrive at their destination.

**Supplementary Table 1:** A list of the main iceberg thermodynamics coefficients and constants.

| **Coefficient** | **Description** | **Units** | **Value** |
| --- | --- | --- | --- |
| $\Gamma_{i}$ | latent heat of fusion of ice | J/kg | 3.33x10^5^ |
| $T_{i}$ | Iceberg temperature | °C | -4 |
| $\alpha$ | Iceberg albedo | dimensionless | 0.7 |
| $k_{a}$ | Thermal conductivity of air (at 10°C) | J/s/m/K | 0.0249 |
| $k_{w}$ | Thermal conductivity of water (at 0°C) | J/s/m/K | 0.563 |
| $\mathcal{v}_{a}$ | kinematic viscosity of air (at 10°C) | m^2^/s | 1.46x10^-5^ |
| $\mathcal{v}_{w}$ | kinematic viscosity of water (at 0°) | m^2^/s | 1.83x10^-6^ |
| $D_{a}$ | thermal diffusivity air (at 0°C) | m^2^/s | 2.16x10^-5^ |
| $D_{w}$ | thermal diffusivity water (at 0°C) | m^2^/s | 1.37x10^-7^ |
| $R$ | Roughness height of the iceberg | m | 0.01 |
| $W_{p}$ | Wave period | s | 6.2 |

**Supplementary Table 2:** Iceberg size distribution used in the control simulation. Note that iceberg length is fixed at 1.6-times iceberg width.

| **Size Class** | **Fraction (%)** | **Width (m)** | **Thickness (m)** |
| --- | --- | --- | --- |
| 1 | 15 | 67 | 80 |
| 2 | 15 | 133 | 160 |
| 3 | 20 | 200 | 240 |
| 4 | 15 | 267 | 320 |
| 5 | 8 | 333 | 360 |
| 6 | 7 | 400 | 360 |
| 7 | 5 | 500 | 360 |
| 8 | 5 | 600 | 360 |
| 9 | 5 | 800 | 360 |
| 10 | 5 | 1000 | 360 |

**Supplementary Table 3:** Initial size of icebergs towed to Cape Town and the United Arab Emirates.

| **Length (m)** | **Width (m)** | **Thickness (m)** |
| --- | --- | --- |
| 200 | 125 | 150 |
| 300 | 187.5 | 150 |
| 400 | 250 | 150 |
| 500 | 312.5 | 150 |
| 600 | 375 | 150 |
| 700 | 437 | 150 |
| 800 | 500 | 150 |
| 900 | 562 | 150 |
| 1000 | 625 | 150 |
| 200 | 125 | 200 |
| 300 | 187.5 | 200 |
| 400 | 250 | 200 |
| 500 | 312.5 | 200 |
| 600 | 375 | 200 |
| 700 | 437 | 200 |
| 800 | 500 | 200 |
| 900 | 562 | 200 |
| 1000 | 625 | 200 |
| 300 | 187.5 | 225 |
| 400 | 250 | 225 |
| 500 | 312.5 | 225 |
| 600 | 375 | 225 |
| 700 | 437 | 225 |
| 800 | 500 | 225 |
| 900 | 562 | 225 |
| 1000 | 625 | 225 |
| 300 | 187.5 | 250 |
| 400 | 250 | 250 |
| 500 | 312.5 | 250 |
| 600 | 375 | 250 |
| 700 | 437 | 250 |
| 800 | 500 | 250 |
| 900 | 562 | 250 |
| 1000 | 625 | 250 |
| 400 | 250 | 300 |
| 500 | 312.5 | 300 |
| 600 | 375 | 300 |
| 700 | 437 | 300 |
| 800 | 500 | 300 |
| 900 | 562 | 300 |
| 1000 | 625 | 300 |
| 400 | 250 | 350 |
| 500 | 312.5 | 350 |
| 600 | 375 | 350 |
| 700 | 437 | 350 |
| 800 | 500 | 350 |
| 900 | 562 | 350 |
| 1000 | 625 | 350 |
| 700 | 437 | 400 |
| 800 | 500 | 400 |
| 900 | 562 | 400 |
| 1000 | 625 | 400 |
| 800 | 500 | 500 |
| 900 | 562 | 500 |
| 1000 | 625 | 500 |
| 1250 | 781.25 | 200 |
| 1250 | 781.25 | 300 |
| 1250 | 781.25 | 400 |
| 1250 | 781.25 | 500 |
| 1250 | 781.25 | 600 |
| 1500 | 937.5 | 200 |
| 1500 | 937.5 | 300 |
| 1500 | 937.5 | 400 |
| 1500 | 937.5 | 500 |
| 1500 | 937.5 | 600 |
| 2000 | 1250 | 200 |
| 2000 | 1250 | 300 |
| 2000 | 1250 | 400 |
| 2000 | 1250 | 500 |
| 2000 | 1250 | 600 |
| 3000 | 1875 | 200 |
| 3000 | 1875 | 300 |
| 3000 | 1875 | 400 |
| 3000 | 1875 | 500 |
| 3000 | 1875 | 600 |
